# Determinants of microalbuminuria among type 2 diabetes mellitus patients in Kuala Selangor district: A cross-sectional study

**DOI:** 10.51866/oa.122

**Published:** 2022-08-25

**Authors:** Nurul Farehah Shahrir, Noor Rafizah Aminah Aziz, Fatimah Lailiza Ahmad, Nor Anizah Muzaid, Farhani Samat, Sharifah Nurul Aida Syed Ghazaili, Nuraini Dolbasir, Nurul Nadia Baharum, Sharmilee T. Ramanathan, Siti Zaharah Abd Rahman, Ap. Sa’aidah Bat, Maznah Sarif, Noor Afiza Ismaal

**Affiliations:** 1 MBBS (UiTM), MPH, DrPH (USM) Pejabat Kesihatan Daerah Kuala, Selangor, Jalan Semarak, Bandar, Melawati, Kuala Selangor, Malaysia. Email: pare87_me@yahoo.com; 2 MBBS (UM), MPH (USM) Pejabat Kesihatan Daerah Kuala, Selangor, Jalan Semarak, Bandar, Melawati, Kuala Selangor, Malaysia.; 3 MD(UKM) Pejabat Kesihatan Daerah Kuala, Selangor, Jalan Semarak, Bandar, Melawati, Kuala Selangor, Malaysia; 4 MD(UKM) MMed(Family Medicine) (UKM) Klinik Kesihatan Kuala Selangor, Jalan Klinik, Bandar Malawati, Kuala, Selangor, Malaysia.; 5 MD(UKM) MMed (Family Medicine) UiTM, Klinik Kesihatan Tanjong karang, Kuala Selangor, Malaysia.; 6 MD(UKM) MMed (Family Medicine) (USM) Klinik Kesihatan Bestari Jaya, Bestari Jaya, Malaysia.; 7 MD (UKM), MMed (Family Medicine) (UM) Klinik Kesihatan Jeram, Jalan Klang-Telok Intan, Jeram, Malaysia.; 8 MBBS (IIUM), MMed (Family Medicine) (UiTM) Klinik Kesihatan Bukit Cherakah, Jalan Rizab Masjid, Jeram, Malaysia.; 9 MBBS (MAHE,MANIPAL)(FRACGP/MAFP) Klinik Kesihatan Ijok, JKR 1087, Jalan 14, Ijok, Batang Berjuntai, Malaysia.; 10 MBBS (CUCMS) Klinik Kesihatan Sg. Tengi Kanan, Jalan Kiai Moid, Tanjong Karang, Malaysia.; 11 MD (USU) Klinik Kesihatan Bukit Cherakah, Jln Rizab Masjid, Kg. Bukit Cherakah, Jeram, Malaysia.; 12 Diploma Kejururawatan, Klinik Kesihatan Bukit Cherakah, Jln Masjid, Jeram, Malaysia.; 13 Diploma Kejururawatan, Klinik kesihatan Sg Tengi Kanan, Jalan Kiai Moid, Tanjung Karang, Malaysia.

**Keywords:** Microalbuminuria, Type 2 diabetes mellitus, HDL, HbAlc, Neuropathy

## Abstract

**Introduction:**

Microalbuminuria presents significant health risks for the progression of endstage renal-failure (ESRF) among type 2 diabetes mellitus (T2DM) patients. This study aims to determine the proportion and associated factors of microalbuminuria among T2DM patients in Kuala Selangor district, Malaysia.

**Method:**

A retrospective cross-sectional study was conducted from December 2020 to February 2021 using secondary data from the National Diabetic Registry (NDR), Malaysia, and reviewed patients’ diabetic records for the year 2020. All T2DM patients aged >18 years who were registered with the NDR in 2020 and fulfilled the inclusion and exclusion criteria were included in the study. Descriptive statistics and multiple logistic regression analysis were performed. Data were analysed using SPSS version 26.0. A total of 343 samples were included in this study for the determination of the proportion of microalbuminuria and its associated factors.

**Results:**

Of 343 respondents, 34.4% had microalbuminuria. HbAlc >7.0% (AdjOR 2.19, 95% CI: 1.35, 3.55, p=0.001), HDL <1.04 mmol/L (AdjOR 2.44, 95% CI: 1.323, 4.52, p=0.004), dyslipidaemia (AdjOR 1.90, 95% CI: 1.03, 3.48, p=0.039), and peripheral neuropathy (AdjOR 3.01, 95% CI: 1.02, 8.93, p=0.047) were significantly associated with microalbuminuria. Conclusion: Microalbuminuria is a modifiable risk factor in preventing the progression of ESRF among T2DM patients. Therefore, identification of factors associated with microalbuminuria among this high-risk group is important to facilitate early screening and prompt treatment to prevent progression of diabetic kidney disease to ESRF.

## Introduction

Globally, the increasing prevalence of type 2 diabetes mellitus (T2DM) is becoming a major public health concern. Worldwide, the number of people affected by diabetes increased from 108 million in 1980 to 422 million in 2014.^1^ Furthermore, from 1980 to 2014, the global prevalence of diabetes in adults increased by 3.8%. The rising prevalence of diabetes mellitus is more pronounced and rapid in low- and middle-income countries than in high-income countries,^2^ including Malaysia. According to the latest National Health Morbidity Survey (2019), the prevalence of diabetes in adults in Malaysia increased from 11.2% in 2011 to 13.4% in 2015, reaching 18.3% in 2019, which is equivalent to approximately 3.9 million adults.^3^ Diabetes is a major cause of blindness, ischemic heart disease (IHD), cerebrovascular accident (CVA), lower limb amputation, and renal failure. Globally, diabetes-related premature mortality increased by 5% between 2000 and 2016, with 1.5 million fatalities directly caused by diabetes in 2019.^4^ Nearly half of all deaths attributable to hyperglycaemia occur before the age of 70 years. According to the World Health Organisation (WHO), diabetes was the ninth leading cause of death in 2019.

Chronic kidney disease (CKD) and end-stage renal disease (ESRD) are becoming more prevalent globally. The global prevalence of CKD ranges between 11% and 13%.^5^ In Malaysia, most recent population-based study reported the prevalence of CKD as 15.48% of the total population in 2018 compared to 9.07% in 2013, with 0.08% of patients having stage 5 CKD or ESRD.^6,7^ Since 2013, the number of Malaysians with ESRD receiving dialysis had more than doubled to 1,059 per million population (pmp) from 415 pmp in 2003.^8^ The increase in ESRD has been largely attributed to the rising prevalence of diabetic nephropathy, which accounts for 58% of new dialysis patients.^9^

The increasing number of patients with ESRD places a significant strain on the healthcare system in terms of human, economic, and social costs. Over a 7-year period, the annual ESRD spending of the public sector increased by 94% from MYR 572 million in 2010 to MYR 1.12 billion in 2016. Total ESRD expenditure increased from 2.95% of total health expenditure in the public sector in 2010 to 4.2% in 2016.^10^

Microalbuminuria (MA) is an important marker of progression to ESRD and an independent predictor of cardiovascular disease (CVD) and mortality across all levels of glomerular filtration rate (GFR).^10^ Without intervention, 20-40% of T2DM patients with MA progress to overt nephropathy and, 20 years later, approximately 20% develop ESRD.^11^ The 2018 Malaysian clinical practice guidelines for the management of CKD in adults recommend screening for MA in T2DM at time of diagnosis followed by yearly screening, with diagnosis of MA requiring two of three abnormal test results. Early diagnosis of MA is important because effective treatments exist to limit the progression of diabetic nephropathy.^12^ Globally, the prevalence of MA among T2DM patients is 36.3% in India,^13^ 44.6% in Kazakhstan,^14^ 33.2% in Saudi Arabia,^15^ 14.2% in Sub-Saharan Africa,^16^ and 32.1% in Singapore.^17^ Meanwhile, in Malaysia, 25.4% of MA was reported among T2DM patients in one tertiary centre in Kelantan.^18^ Among the factors associated with MA in T2DM are increasing age,^19^ female gender,^15^ ethnicity,^20^ obesity, poorly controlled blood pressure,^19^ neuropathy, and macrovascular complications.^15^

There is limited published literature on the prevalence of MA and its associated factors in Malaysia. To date, there was one published study on MA among T2DM patients in a tertiary centre on the East Coast in which the findings might differ from the population in Kuala Selangor due to different population demography and socio-cultural aspects.^18^ In addition, the increasing prevalence of diabetes in Malaysia will give rise to diabetic complications, particularly diabetic nephropathy and ESRF, resulting increasing healthcare costs. This will eventually lead to morbidity and premature mortality. Diabetic nephropathy is usually asymptomatic until the late stage; therefore, early screening and detection of MA is crucial to prevent its progression. This is in line with the United Nations third Sustainable Development Goal on health and wellbeing, target 3.4: to reduce premature mortality from noncommunicable diseases (NCDs) by one-third between 2015 and 2030. This is in addition to the WHO target of reducing premature deaths from NCDs by 25% between 2010 and 2025. Thus, the aim of this study is to determine the proportion and associated factors of MA among T2DM patients. We believe that identifying the determinants of MA among T2DM patients will help providers to identify patients who are at risk of developing MA for effective intervention. This study will also help healthcare providers to continuously improve the services they deliver.

## Methods

### Study type and design

This study applied a cross-sectional design based on retrospective data review between December 2020 and February 2021 in Klinik Kesihatan Bukit Cherakah and Klinik Kesihatan Sungai Tengi Kanan.

Secondary data were collected from the National Diabetic Registry (NDR) and review of patients’ records. The NDR is an online registry set up by the Ministry of Health in 2009 for surveillance purpose of the diabetic population in Malaysia to monitor the quality of care in patients. Kuala Selangor is one of the districts in Selangor state bounded by Sabak Bernam in the north, Hulu Selangor and Gombak in the west, Petaling in the southwest, and Klang in the south. It has a total of nine mukims. Based on the census in 2010, it had an estimated population of 209,590. The majority of the population were Malay (74.9%) followed by Indian (15.4%) and Chinese (9.5%); 65.4% of the population were aged between 15 and 64 years.

### Study participants

The reference populations were all active T2DM patients in Kuala Selangor district in 2020, and the study samples were all active T2DM patients registered with the NDR who attended Bukit Cherakah and SgTengi Kanan health clinics in 2020 and fulfilled the inclusion and exclusion criteria. In this study, the inclusion criteria were Malaysian citizen, aged ≥18 years, who had performed a urine microalbumin investigation in 2020. The exclusion criteria were T2DM patients who were pregnant or had comorbidities resulting in proteinuria, such as congestive cardiac failure, overt proteinuria, kidney disease other than diabetic, and stage 4 CKD and ESRF as these additional risk factors would be confounding factors during the analysis.

The sample size was calculated for each variable of associated factors for MA using Power and Sample Size calculation to compare two independent proportions. The largest estimated sample for each group was 345 based on a probability of exposure of associated factors among normoalbuminuria from a literature review,^21^ a power of 80% (0.8), alpha of 0.05, Po : 0.2 Pi : 0.34, M : 1, the calculated sample size was 314 patients. Allowing for a 10% dropout rate, a final sample size of 345 was used.

### Data collection and analysis

Simple random sampling was applied for sample selection from a list generated from the NDR from each of the health clinics based on proportionate sampling calculated based on the annual active patients registered at each clinic. All data fulfilling the inclusion and exclusion criteria were analysed. Data were collected from both the NDR and patients’ diabetic books. The retrieved information comprised sociodemographic data (age, sex, race), anthropometric measurements (weight, height, body mass index [BMI]), duration of diabetes, lifestyle (smoking status), laboratory results (HbA1c, low-density lipoprotein [LDL], creatinine), usage of ACEi/ARBs, comorbidities (hypertension, hyperlipidaemia, gout), diabetic complications (microvascular: diabetic retinopathy/premature cataract, CKD, and peripheral neuropathy; macrovascular: CVD, CVA, and PVD), and staff factors.

MA is considered positive when the urinary albumin-to-creatinine ratio (ACR) is 30-300 mg/g creatinine in two of three tests performed within a 3-6-month period on a spot urine sample.^22^ However, in this study, the test was performed annually for each patient due to unforeseen circumstances. Measurement of urine creatinine was performed using Siemens Microalbumin Test Strip, and measurement of urine microalbumin was performed using Siemens Microalbumin urine analyser. The data were extracted from the patients’ diabetic record books.

The independent variables were categorised according to ethnicity, which was divided into four groups (Malay, Chinese, Indian, and other) and smoking status (smoking, non-smoking, or quit ≥6 months). BMI was calculated by weight in kilograms divided by height in meters squared and was classified into two categories (normoweight and others) based on the 2003 Malaysian Clinical Practice Guidelines on Management of Obesity cutoff point. Laboratory investigation classified HbA1c ≤7.0% or >7.0%,^23^ while high-density lipoprotein (HDL) was classified as ≥1.04 mmol/L or <1.04 mmol/L.^24^ The comorbidities, usage of ACEi/ARBs, and diabetic complications were categorised as ‘yes’ or ‘no’ and were extracted from the diabetic records. Finally, staff factors, including medical lab technicians (MLTs) who performed urine microalbumin tests not according to the protocol (i.e., performed on a patient with positive urine dipstick for proteinuria) were categorised as ‘yes’ or ‘no’.

### Statistical analysis

The statistical package for social sciences (SPSS) version 26.0 software was used for data entry and analysis. Descriptive statistics with mean and standard deviation (SD), frequency, and percentages were calculated. Simple and multiple logistic regression analyses were used to determine factors associated with MA. Multiple logistic regression using the forward stepwise method was performed. In this study, the single dichotomous outcome was coded as 0 for non-MA and 1 for MA. Univariable analysis was performed to select the variables into multiple logistic regression analysis, and only variables with p-value < 0.25 or clinical important were selected. Multicollinearity between different predictor variables was checked using a variance inflation factor (VIF). All possible two-way interaction terms between significant variables were checked one at a time. The final model was determined where the adjusted odds ratio was estimated with a 95% confidence interval (CI). A p-value of less than 0.05 was considered statistically significant.

### Ethical approval

Ethical approval for this study was obtained from the National Medical Research Register Ministry of Health (NMRR-20-2087-56369).

## Results

### Proportion of study population

The total sample retrieved from the registry following the inclusion and exclusion criteria was 343, with 2 (0.16%) participants excluded due to having CKD stage 4.

There were 118 (34.4%) T2DM patients with MA in this study.

**Table 1:** Proportion of T2DM patients with microalbuminuria (n=343).

Variable	n (%)	95% CI
*Microalbuminuria*		
No	225 (65.6)	0.65, 0.68
Yes	118 (34.4)	0.33, 0.35

### Type 2 diabetes mellitus respondents’ characteristics

The respondents’ characteristics are presented in **Table 2**. The mean age was 61.0 (9.8), the majority of the respondents were female (74.1%), of Malay ethnicity (97.4%), and non-smokers (95.3%). Most of the respondents were obese (74.1%) and had hypertension (93.0%) and dyslipidaemia (77.8%). Only 3.5% of the study population had IHD and gout respectively; 0.9% had CVA and none had PVD. In term of microvascular diabetic complications, 4.4% of the participants had retinopathy and neuropathy respectively. Almost half (44.6%) of the participants had stage 2 diabetic CKD and 81.3 % were on ACEi/ARBs. Regarding laboratory parameters, most of the respondents had HDL ≥1.04 mmol/L (84.0%) and HbA1c ≤7.0% (66.8%). Only 37.6% of the patients with MA had urine microalbumin investigations performed by MLTs not according to protocol.

**Table 2:** Characteristics of type 2 diabetes mellitus patient in PKD Kuala Selangor (n=343).

Variable	Total Mean (SD), n (%)	Outcome (n, %)	
		Normal (n=225)	Microalbuminuria (n=118)
*Age (years)*	61.0 (9.8)	61.1 (8.9)	60.7 (11.0)
*Gender*			
Female	254 (74.1)	168 (74.7)	86 (72.9)
Male	89 (25.9)	57 (25.3)	32 (27.1)
*Ethnicity*			
Malay	334 (97.4)	222 (98.7)	112 (94.9)
Chinese	9 (2.6)	3 (1.3)	6 (5.1)
*Klinik kesihatan*			
Bukit Cherakah	139 (40.5)	85 (37.8)	54 (45.8)
Sg Tengi Kanan	204 (59.5)	140 (62.2)	64 (54.2)
*Duration of diabetes (years)*	7.87 (5.3)	7.93 (5.7)	7.77 (4.5)
*Smoking status*			
Non-smoking	327 (95.3)	217 (96.4)	110 (93.2)
Smoking	16 (4.7)	8 (3.6)	8 (6.8)
*BMI (kg/m^2^)*			
Normoweight	38 (11.1)	25 (11.1)	13 (11.0)
Underweight	5 (1.5)	4 (1.8)	1 (0.8)
Overweight	46 (13.4)	29 (12.9)	17 (14.4)
Obese	254 (74.1)	167 (74.2)	87 (73.7)
*Comorbidities*			
Hypertension No	24 (7.0)	16 (7.1)	8 (6.8)
Yes	319 (93.0)	209 (92.9)	110 (93.2)
Dyslipidaemia No	76 (22.2)	58 (25.8)	18 (15.3)
Yes	267 (77.8)	167 (74.2)	100 (91.9)
Gout			
No	331 (96.5)	217 (96.4)	114 (96.6)
Yes	12 (3.5)	8 (3.6)	4 (3.4)
*Macrovascular complications*			
IHD No Yes	331 (96.5) 12 (3.5)	218 (96.9) 7 (3.1)	113 (95.8) 5 (4.2)
CVA			
No	340 (99.1)	223 (99.1)	117 (99.2)
Yes	3 (0.9)	2 (0.9)	1 (0.8)
PVD			
No	343 (100.0)	225 (100.0)	118 (100.0)
Yes	0 (0.0)	0 (0.0)	0 (0.0)
*Microvascular complications*			
Retinopathy			
No	328 (95.6)	214 (95.1)	114 (96.6)
Yes	15 (4.4)	11 (4.9)	4 (3.4)
Neuropathy			
No	328 (95.6)	219 (97.3)	109 (92.4)
Yes	15 (4.4)	6 (2.7)	9 (7.6)
Chronic kidney disease			
Stage 1	92 (26.8)	61 (27.1)	31 (26.3)
Stage 2	153 (44.6)	103 (45.8)	50 (42.4)
Stage 3a	76 (22.2)	50 (22.2)	26 (22.0)
Stage 3 b	22 (6.4)	11 (4.9)	11 (9.3)
*Medications*			
ACEi/ARB			
No	64 (18.7)	44 (19.6)	20 (16.9)
Yes	279 (81.3)	181 (80.4)	98 (83.1)
**Lab investigations**			
LDL (mmol/L)	2.71 (0.9)	2.70 (0.9)	2.71 (1.0)
HDL (mmol/L)			
≥1.04	288 (84.0)	196 (87.1)	92 (78.0)
<1.04	55 (16.0)	29 (12.9)	26 (22.0)
HbAlc			
≤7.0%	229 (66.8)	163 (72.4)	66 (55.9)
>7.0%	114 (33.2)	62 (27.6)	52 (44.1)
Creatinine (μmol/L)	85.2 (26.8)	83.0 (23.4)	89.3 (26.9)
*MLT (staff) performing test not according to protocol*			
No	214 (62.4)	148 (65.8)	66 (55.9)
Yes	129 (37.6)	77 (34.2)	52 (44.1)

### Factors associated with microalbuminuria

Simple logistic regression showed that ethnicity, dyslipidaemia, neuropathy, HDL, HbAlc, creatinine level, and staff (MLT) were significantly associated with MA at p<0.25. Chinese ethnicity (crude OR 3.96, 95% CI: 0.97, 16.15, p=0.055), dyslipidaemia (crude OR 1.93, 95% CI: 1.08, 3.46, p=0.027), and neuropathy (crude OR 3.01, 95% CI: 1.05, 8.68, p=0.04l). In addition, HDL <1.04 mmol/L (crude OR 1.97, 95% CI: 1.09, 3.58, p=0.030), HbA1c >7.0% (crude OR 1.20, 95% CI: 1.07, 1.34, p=0.002), creatinine (crude OR 1.01, 95% CI: 1.00, 1.02, p=0.026), and MLT staff performing the test not according to protocol (crude OR 1.51, 95% CI: 0.96, 2.39, p=0.074) were found to be significantly associated with MA. No significant association between age, gender, duration of diabetes, smoking status, BMI, gout, macrovascular complications, retinopathy, usage of ACEi/ARBs, and LDL level with MA was observed. The results are summarised in **Table 3**

**Table 3:** Simple logistic regression of factors associated with microalbuminuria (n=343).

Variable	Regression coefficient B	Crude OR (95% CI)	Wald statistic (df)	p-value
*Age (years)*	-0.04 (0.01)	1.00 (0.97, 1.02)	0.14 (1)	0.709
*Gender*				
Female		1.00		
Male	0.09 (0.26)	1.10 (0.66, 1.82)	0.13 (1)	0.720
*Ethnicity*				
Malay		1.00		
Chinese	1.38 (0.72)	3.96 (0.97, 16.15)	3.70 (1)	0.055
*Duration ofdiabetes (years)*	-0.01 (0.02)	0.99 (0.95, 1.04)	0.07 (1)	0.795
*Smoking status*				
Non-smoking		1.00		
Smoking	0.68 (0.51)	1.97 (0.72, 5.40)	1.75 (1)	0.186
*BMI (kg/m^2^)*				
Normoweight		1.00		
Other	-0.59 (0.26)	0.93 (0.55, 1.55)	0.09 (1)	0.771
*Comorbidities*				
Hypertension No Yes	0.05 (0.45)	1.00 1.05 (0.44, 2.54)	0.01 (1)	0.909
Dyslipidaemia No Yes	0.66 (0.30)	1.00 1.93 (1.08, 3.46)	4.87 (1)	**0.027**
Gout No Yes	-0.05 (0.62)	1.00 0.95 (0.28, 3.23)	0.00 (1)	0.937
*Complications*				
IHD No Yes	0.32 (0.60)	1.00 1.38 (0.43, 4.44)	0.29 (1)	0.591
CVA No Yes	-0.05 (1.23)	1.00 0.95 (0.09, 10.62)	0.00 (1)	0.969
Retinopathy No Yes	-0.32 (0.60)	1.00 0.68 (0.21, 2.20)	0.41 (1)	0.521
Neuropathy No Yes	1.10 (0.54)	1.00 3.01 (1.05, 8.68)	4.18 (1)	0.041
*Medications*				
ACEi/ARB Yes No	0.18 (0.30)	1.00 1.19 (0.67, 2.13)	0.35 (1)	0.556
*Lab investigations*				
LDL (mmol/L)	0.00 (0.12)	1.00 (0.79, 1.28)	0.00 (1)	0.980
HDL (mmol/L) ≥1.04 <1.04	0.68 (0.31)	1.00 1.97 (1.09, 3.58)	4.96 (1)	**0.030**
HbA1c ≤7.0% >7.0%	0.18 (0.06)	1.00 1.20 (1.07, 1.34)	10.07 (1)	**0.002**
Creatinine (μmol/L)	0.01 (0.01)	1.01 (1.00, 1.02)	4.98 (1)	**0.026**
*MLT (staff)*				
No		1.00		
Yes	0.42 (0.23)	1.51 (0.96, 2.39)	3.18 (1)	**0.074**

Multiple logistic regression analysis revealed that HbA1c level, HDL level, dyslipidaemia, and neuropathy were significantly associated with MA (**Table 4**). Respondents with HbA1c >7.0% had 2.19 times the odds of having MA compared with HbA1c ≤7.0% (95% CI: 1.35, 3.55, p=0.001) when adjusted for HDL level, dyslipidaemia, and neuropathy. Respondents with HDL <1.04 mmol/L had 2.44 times the odds of having MA compared to participants with HDL ≥1.04 mmol/L (95% CI: 1.32, 4.52, p=0.005). In addition, respondents with dyslipidaemia had 1.90 times the odds of having MA compared with those without hyperlipidaemia when adjusted for HbA1c, HDL, and neuropathy (95% CI: 1.03, 3.48, p=0.039). Participants with neuropathy had 3.01 times the odds of having MA compared with those without neuropathy when adjusted for HbA1c, HDL, and dyslipidaemia (95% CI: 1.02, 8.93, p=0.047).

**Table 4:** Multiple logistic regression of factors associated with microalbuminuria (n=346).

Variable	Regression coefficient B	Adjusted OR (95% Cl)	Wald statistic (df)	*p*-value
*Lab investigations*				
IlbAlC ≤7.0% >7.0%	0.78 (0.25)	1.00 2.19 (1.35, 3.55)	10.11 (1)	0.001
HDL (mmol/L) ≥1.04 <1.04	0.89 (0.32)	1.00 2.44 (1.32,4.52)	8.04 (1)	0.005
*Comorbidities*				
Dyslipidacmia				
No		1.00		
Yes	0.64 (0.31)	1.90 (1.03, 3.48)	4.28(1)	0.039
*Complications*				
Neuropatdy				
No				
Yes	1.10(0.56)	3.01 (1.02, 8.93)	3.95 (1)	0.047

Constant = -1.642 Enter LR method was applied No multicollinearity and no interaction Hosmer—Lemeshow test, p-value <0.001 Classification table 68.5 % correctly classified Area under Receiver Operating Characteristics (ROC) curve was 64.5%

## Discussion

### Proportion of microalbuminuria

Previous studies reported marked variation in the prevalence of MA across countries. The proportion of MA in our T2DM patients was 34.4% and was comparable with the previous MA prevalence study on hypertensive T2DM patients in Malaysia in 2006 (39.7%) and a study in India (36.3%).^13,21^ The prevalence of MA in this study was much lower than the study among 289 T2DM patients in a tertiary clinic in Bostwana (44.6%) and Korea (56.5%) in a multicenter epidemiological study across 10 countries in Asia^14,25^; but higher compared to the prevalence of MA in Singapore (14.2%) Canada (15%) and the U.K. (24.9%).^26-28^ This variation in prevalence can be attributed to factors such as differences in study populations, ethnic susceptibility, definition of MA, and method of assessment of MA between those studies. Adler et.al reported in the U.K. Prospective Diabetes Study (UKPDS 64) that the annual transition rate of MA to macroalbuminuria was 2.8%, and 2.3% of macroalbuminuria progressed to elevated plasma creatinine or required renal replacement therapy.^28^

### Factors associated with microalbuminuria

In our study, diabetic patients who had higher HbA1c level were significantly associated with MA. In line with this finding, Lee and Tang^26^ found that poorer diabetic control was associated with an almost two-fold increased risk for MA (OR 1.88, 95% CI: 1.26, 2.7). Other studies reported similar results on the relationship between poorer diabetic control and MA.^29,30^ Excess intracellular glucose has been demonstrated to stimulate cellular signalling pathways, including the diacylglycerol (DAG)-protein kinase C (PKC) pathway, polyol pathway, hexosamine pathway, advanced glycation end-products (AGEs), and oxidative stress, all of which contribute to glomerulosclerosis.^31^ In addition, Rho-kinase, a small GTPase-binding protein effector, has been linked to ultrastructural damage by inducing endothelial dysfunction, excessive extracellular matrix (ECM) production in the mesangial cells, podocyte abnormalities, and tubulointerstitial fibrosis, resulting in diabetic nephropathy and MA.^32^

Our findings suggest that having dyslipidaemia and lower HDL levels were significantly associated with MA. This is supported by Sun, Xiao,^33^ who reported an inverse association between MA and HDL after adjusting for blood pressure, age, gender, HbA1c, BMI, total cholesterol, triglycerides, LDL, duration of diabetes, smoking, and medications. Furthermore, insulin has been demonstrated to have a key role in the formation of ApoA-I, a major HDL apolipoprotein.^34^ Therefore, the reduction in insulin action is potentially involved in the low HDL levels observed in T2DM. Moreover, the substantially impaired anti-inflammatory capacity of HDL in T2DM contributes to an increased risk of atherosclerosis.^35^ Moreover in diabetic conditions, dyslipidaemia stimulates macrophage infiltration and excessive ECM production in the glomeruli, resulting in diabetic nephropathy.^32^ Besides, Sasaki et.al found a substantial reduction in urine albumin excretion among diabetic patients receiving lipid-lowering therapy, corroborating the findings.^36^ In contrast, Efundem and Molefe-Baikai et.al found no significant difference between dyslipidaemia, HDL level, and MA.^14,16^ The observed differences in the association between HDL level and MA may be attributed to differences in the cholesterol measurement used in those studies.

Our data suggest a significant association between peripheral neuropathy and MA. There was a 4.9% higher proportion of peripheral neuropathy among the MA group than the normoalbuminuria group. Similar findings were reported in a prospective cohort study in Birmingham in which peripheral neuropathy was independently associated with MA in addition to a local study in Kelantan.^18,37^ Patel et. al reported that those with peripheral neuropathy had a higher albumin excretion rate (15.2±6.3 micrograms/min) than participants with normoalbuminuria.^38^ These findings are consistent with the presence of a microvascular component in the pathogenesis of diabetic neuropathy. Although the staff factor is only significant at a univariate level, our findings highlight the importance of staff education and continuous assessment on performing urine microalbumin analysis, as affects the prevalence and the early identification of patients with MA and their referral for prompt treatment. The effect of the staff factor, techniques on variability, and reliability of test results has been demonstrated in other studies.^39,40^ Finally, hypertension, IHD, CVA, PVD, and smoking status were not significantly associated with MA in our study; however the results should be interpretated with caution due to the small sample size of each subgroup.

Despite its associated complications, T2DM is a condition that is mostly preventable and treated. Based on our findings, in addition to the large proportion of T2DM patients who are obese (74.1%) in this study and in Malaysia as a whole, primary prevention strategies should target high-risk groups, such as obese patients, who have been identified by the WHO,^1^ as a major contributor to the global rise in diabetic prevalence. Early weight intervention, diabetic screening for early diagnosis, and timely treatment to avoid diabetic progression should all be part of these prevention efforts.

These prevention strategies should include early weight intervention, diabetic screening for early diagnosis, and prompt treatment to prevent diabetic progression. For secondary prevention, MA screening should be performed annually in all patients with T2DM, especially among those with HbA1c >7.0%, HDL <1.04 mmol/L, dyslipidaemia, and peripheral neuropathy, as early treatment with CV risk reduction measures is crucial. The primary strategy is to halt the progression of renal disease via prompt treatment of diabetes and other comorbidities, including dyslipidaemia. While MA testing is crucial, ensuring proper procedure by performing staff according to protocols should be ensured through periodic audits.

To our knowledge, this is the first population-based study in Malaysia that showed the association of HbA1c >7.0%, HDL <1.04 mmol/L, dyslipidaemia, and peripheral neuropathy with MA in T2DM patients. Nevertheless, this study is not without limitations. First, due to the nature of this study, we could not establish causality. Second, only two health clinics were involved in this study, which limits its generalisability.

Nevertheless, an adequate sample size was reached based on the sample size calculation to achieved 80% power of the study. Third, due to the nature of the secondary data, information on comorbidities, such as IHD, CVA, PVD, hypertension, and smoking status, may have changed and may not have been updated from the initial records. Fourth, most patients with complications were treated in hospitals, limiting the number of patients with complications in this study. Therefore, future studies should include patients in hospitals, health clinics, and the community so that the true burden of MA can be appreciated.

In conclusion, the proportion of MA in Kuala Selangor district was 34.4%. The factors found to be associated with MA in this study were poorer diabetic control, lower HDL level, dyslipidaemia, and peripheral neuropathy. MA is not only a risk factor for ESRF in diabetes, but it is also an important marker of mortality in the diabetic population. Early detection of MA among high-risk diabetes patients and early treatment of MA would prevent the progression of diabetic nephropathy, thereby preventing premature death.
